# Alkaline thermostable pectinase enzyme from *Aspergillus niger* strain MCAS2 isolated from Manaslu Conservation Area, Gorkha, Nepal

**DOI:** 10.1186/s40064-015-1286-y

**Published:** 2015-09-09

**Authors:** Bhim Prakash Khatri, Tribikram Bhattarai, Sangita Shrestha, Jyoti Maharjan

**Affiliations:** Nepal Academy of Science and Technology, Khumaltar, P.O.Box. No. 3323, Lalitpur, Nepal; Central Department of Biotechnology, Tribhuvan University, Kirtipur, Nepal

**Keywords:** Pectinase, *Aspergillus niger* strain MCAS2, Manaslu Conservation Area (MCA)

## Abstract

Pectinase enzymes are one of the commercially important enzymes having great potential in various industries especially in food industry. Pectinases accounts for 25 % of global food enzymes produced and their market is increasing day by day. Therefore, the exploration of microorganism with novel characteristics has always been the focus of the research. Microorganism dwelling in unique habitat may possess unique characteristics. As such, a pectinase producing fungus *Aspergillus niger* strain MCAS2 was isolated from soil of Manaslu Conservation Area (MCA), Gorkha, Nepal. The optimum production of pectinase enzyme was observed at 48 h of fermentation. The pectinase enzyme was partially purified by cold acetone treatment followed by Sephadex G-75 gel filtration chromatography. The partially purified enzyme exhibited maximum activity 60 U/mg which was almost 8.5-fold higher than the crude pectinase. The approximate molecular weight of the enzyme was found to be 66 kDa as observed from SDS-PAGE. The pectinase enzyme was active at broad range of temperature (30–70 °C) and pH (6.2–9.2). Optimum temperature and pH of the pectinase enzyme were 50 °C and 8.2 respectively. The enzyme was stable up to 70 °C and about 82 % of pectinase activity was still observed at 100 °C. The thermostable and alkaline nature of this pectinase can meet the demand of various industrial processes like paper and pulp industry, in textile industry, fruit juice industry, plant tissue maceration and wastewater treatment. In addition, the effect of different metal ions on pectinase activity was also studied.

## Background

Pectinase enzymes commonly represent a group of enzymes that involves in degradation of pectin. Pectins are the high molecular weight polysaccharides, primarily composed of α-1 → 4 linked D galacturonic acid residues with a small number of rhamnose residues in the main chain and arabinose, galactose and xylose on its side chain (Rangarajan et al. 2010). Pectin is found in middle lamella and primary cell wall of higher plants in the form of calcium pectate and magnesium pectate (Favela-Torress et al. 2003). It gives rigidity to plant. The amount of pectic substances present in plant materials varies (0.5–4.0 % of fresh weight plant material) depending upon the source of plant materials (Jayani et al. 2005; Sakai et al. 1993). During the developing stage of plants, the structure of these pectic substances is altered due to the activity of pectinases. These enzymes facilitate the cell wall extension and softening of some tissues during maturation of the part of plants such as fruits.

Enzymes are important biocatalyst for various industrial and biotechnological purposes and produced by microorganisms, animal and plant. They can work in many adverse condition compared to chemical catalyst. As such many microbial enzymes are being used for biotechnological and industrial purposes. Microorganisms are preferred as a source of enzyme because of their short life span, high productivity rate, cost effective, and also free of harmful chemicals that are found in enzyme from plant and animal source (Chaplin and Bucke 1990). Fifty percent of available enzymes are originated from fungi and yeast; 35 % from bacteria, while the remaining 15 % are either of plant or animal origin. Filamentous microorganisms are most widely used for pectinase production (Soares et al. 1999).

Pectinase enzymes are classified into polygalcturonase(PG), pectinesterase(PE), and pectin lyase(PL) based on their mode of action on the substrate (Jayani et al. 2005). Pectinase enzymes are extensively used in an industrial sector especially in food industry i.e. fruit juice extraction, coffee and tea fermentation, oil extraction, improvement of chromaticity and stability of red wine (Jayani et al. 2005). Besides food industry; pectinases are widely used in textile, paper and pulp industries, waste-water treatment (Solbak et al. 2005; Ahlawat et al. 2014). More recently, the enzyme has been used with cellulose enzyme for the production bioethanol from lignocellulosic biomass (Li et al. 2014).

Depending upon the pH requirement for optimum enzymatic activity, pectinase enzyme is also classified into acidic and alkaline pectinase. Acidic pectinases are useful in extraction, clarification and liquefaction of fruit juices (Kaur et al. 2004) and wines (Favela-Torres et al. 2005). Whereas, alkaline pectinases are widely used in the fabric industry, pulp and paper industry and in improving the quality of black tea (Sharma and Satyanarayan 2004; Favela-Torres et al. 2005).

Pectinase enzymes are produced by many microorganism such as bacteria (Takao et al. 2000; Kapoor et al. 2000; Hayashi et al. 1997), fungi (Patil and Dayanand 2006; Huang and Mahoney 1999), yeast (Blanco et al. 1999), and some actinomycete too (Bruhlman et al. 1994). Few pectinase enzymes also have been reported from agro-waste sources like mango peels (Mehrnoush et al. 2012) and Patiya peels (Zohdi and Mehrnoush 2013). Microbial production of pectinases has been extensively studied (Favela-Torres et al. 2006). In practice, the majority of commercially available microbial pectinase comes mainly from fungal source (Singh et al. 1999) of which *Aspergillus* species, predominate. The pectinase enzyme accounts for about 25 % of worldwide sales of industrial enzymes (Prathyusha and Suneetha 2011). Because of its potential application in biotechnology and industry, the demand for commercial pectinase with high stability and novel characteristics to overcome the limitation of existing commercial pectinase is increasing. As such, researchers have focused their attention towards the exploration of new microbial isolates with desirable biochemical and physicochemical characteristics and a low cost production (Silva et al. 2002; Malvessi and Silveira 2004; Phutela et al. 2005). There are many reports on pectinase enzyme from microorganism isolated from different geographical location and sources especially from agro/industrial waste or spoiled fruits. In the context of Nepal, very few studies have been carried out on the microbial pectinase (Gewali et al. 1994, 1997, 2007). In this regard, we attempted to isolate the fungal strain from highlands because the microorganism dwelling in unique habitat may possess unique characteristics. Considering the importance and application of pectinase enzyme the present study aims to characterize the pectinase enzyme produced by a newly isolated soil fungus, *Aspergillus niger* strain MCAS2 from the altitude of 3500 m, Manaslu Conservation Area (MCA), Gorkha district of Nepal.

## Methods

### Isolation of fungal species

The fungi were isolated from soil collected from the altitude of 3500, Manaslu Conservation area, Gorkha by soil dilution plate and soil plate method (Phutela et al. 2005). Morphologically and microscopically different colonies were picked up and subcultured. The spores of pure culture were maintained in potato dextrose agar (PDA) medium in test tubes sealed with parafilm and stored at 4 °C for further use.

### Screening of crude pectinase enzymes

The isolated fungal spores were aseptically inoculated into separate flasks containing Potato Dextrose (PD) broth and incubated at 30 °C and 200 rpm for 14 days. The mycelial mass and cell debris were removed by centrifugation at 4000 rpm for 20 min (FISONS centrifuge, Centaur-2, England). The supernatant thus obtained was used as the crude pectinase enzyme. Cup plate method was used for the screening of pectinase enzyme. The supernatant was added on a PDA media supplemented with 0.5 % pectin substrates and incubated at 37 °C f or 48 h. The plates were stained with 0.1 % iodine solution and clear zone formed around the hole was observed.

### Production and purification of pectinase enzyme

The fungus was grown on defined liquid media as described by Bhardwaj (2010) containing 1 % pure Pectin, 0.1 % (NH_4_)_2_SO_4_, 0.6 % K_2_HPO_4_, 0.2 % KH_2_PO_4_, MgSO_4_·7H_2_O, pH 6.0 and incubated at 30 °C. After 48 h, the cell debris was removed by using Whatman No. 1 filter paper. The crude enzyme (filtrate) was mixed with three volumes of ice cold acetone and allowed to stand for 15 min in ice cold condition (Rajendran et al. 2011).The entire content was centrifuged at 4000 rpm for 20 min. The supernatant was discarded. The precipitate was dissolved in minimum volume of sodium acetate buffer (0.1 M, pH 4.2) and further subjected to Sephadex G-75 (35 × 1.5 cm, bed volume 12–15 ml/g, Sigma company) as per the standard method (Keller et al. 2006) with certain modifications. The molecular weight and the degree of purity were determined by sodium dodecyl sulfate–polyacrylamide gel electrophoresis (SDSPAGE) (Sigma, USA) using the wide range protein molecular weight marker (GeNei, India) according to Laemmli (1970). The protein marker contains myosin (205 kDa), Phosphorylase B (97.4 kDa), Bovine Serum Albumin (66 kDa), Ovalbumin (43 kDa), Carbonic Anhydrase (29 kDa), Soybean Trypsin Inhibitor (20.1 kDa), and Lysozyme (14.3 kDa). Protein bands are visualized using coomassie brilliant blue dye.

### Pectinase assay

The Pectinase activity of partially purified enzyme was assayed by using 3, 5-dinitrosalicylic acid (DNS) as described by (Miller 1959). The reaction mixture contained 1 ml of 0.5 % pectin, 0.5 ml sodium acetate buffer (0.1 M, pH 4.2) and 0.5 ml enzyme. The reaction mixture was incubated at 30 °C in water bath (JulaboLabortechnik GMBH, Germany, and Model-TW12) for 10 min. After 10 min, 2 ml of DNS reagent (Sigma, USA) were added and boiled for 15 min. After cooling, the absorbance was measured in spectrophotometer at 575 nm (6715UV/vis. Spectrophotometer, JENWAY, UK). The reaction mixture containing 1 ml of 0.5 % pectin, 1 ml of sodium acetate buffer (0.1 M, pH 4.2) and 2 ml of DNS reagent was used as a control. The standard curve was simultaneously prepared for reducing sugars with galacturonic acid. One unit of pectinase activity was defined as the amount of enzyme needed to catalyze the reaction 1 mg of galacturonic acid per hour under analysis condition.

### Determination of protein

The concentration of soluble protein was estimated by Biuret method (Gornall et al. 1949) using bovine serum albumin (BSA) as the standard. Specific activity of an enzyme was defined as enzyme units per mg protein.

### Characterization of partially purified pectinase

For the characterization, partially purified pectinase enzyme was used. To determine the optimum temperature of pectinase enzyme, the purified enzyme (5 units) was incubated in sodium acetate buffer pH 4.2 in presence of 0.5 % pectin at different temperature ranging from 30 to 100 °C. To study the effect of pH on enzyme activity, the reaction was carried out using 5 units enzyme and 0.5 % pectin in different buffer system: Sodium acetate buffer (3.2–6.2), sodium phosphate buffer (7.2–8.2), and 0.1 M sodium borate (9.2–10.2) at 30 °C for 10 min. In the same way, the effect of substrate concentration on pectinase activity was carried out by using 0.5–3.0 % pectin in the reaction mixture. The influence of incubation time was measured by incubating the reaction mixture containing 0.5 % pectin in sodium acetate buffer pH 4.2, 5 units enzyme at fixed 30 °C over the period of 1 h at the interval of 10 min. The effects of metal ions on the pectinase activity were determined in the presence of 5 mM Na^+^, Mg^+2^, Pb^+2^, Ba^+2^, Zn^+2^, Ca^+2,^ Cd^+2^ and Fe^+3^ in the reaction mixture.

## Result

### Screening of isolates

**Fig. 1:**
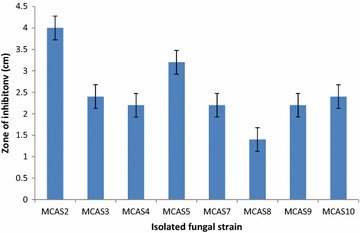
Pectinase activity of different isolated fungal strains

Ten different fungi were isolated from soil samples collected from Manaslu Conservation Area, Gorkha, by using spread plate and soil dilution method. Out of ten isolate, only eight isolates were found pectinase producer. Among them, strain MCAS2 showed the highest pectinase activity and thus selected for the production and characterization of pectinase enzyme. Two isolates, MCAS1 and MCAS6 did not show any pectinase activity (Fig. 1). Based on the colony morphology, microscopic observation and ITS nucleotide sequence homology, the isolate MCAS2 was identified as *Aspergillus niger* strain MCAS2. The Gene Bank accession no for ITS sequence of *Aspergillus niger* strain MCAS2 is KM103363.

### Production and purification of enzyme

**Fig. 2:**
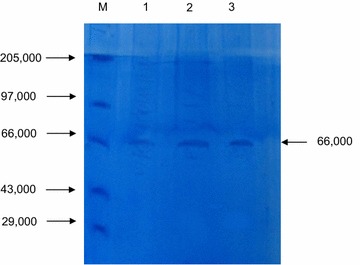
SDS-PAGE of Pectinase enzyme from isolated *Aspergillus niger* strain MCAS2. *Lane M* protein marker in Dalton, *Lane 1* crude pectinase, *Lane 2* partially purified pectinase and *Lane 3* Sephadex G-75 purified pectinase

The production of pectinase was carried out in defined liquid medium. The crude enzyme was harvested from 48 h old culture and acetone precipitated which was followed by gel filtration on Sephadex G-75 column chromatography. Table 1 shows purification summary for pectinase enzyme. It showed that the enzyme was purified 1.2-fold with specific activity of 8.33 U/mg by using cold acetone purification method and 8.5-folds with specific activity of 60 U/mg by using Sephadex method. The apparent molecular weight of pectinase enzyme was found to be 66 kDa as revealed form SDS-PGE analysis (Fig. 2).

**Table 1:** Overall scheme of purification and activity of pectinase from *Aspergillus niger* strain MCAS2

Phase	Volume (ml)	Total activity (U)	Protein (mg/ml)	Protein total (mg)	Specific activity (U/mg)	Yield (%)	Purification fold (X)
Crude extract	100	370	0.525	52.5	7.05	100	1
Acetone ppt.	7	70	1.2	8.4	8.33	19	1.2
Sephadex G-75	3	9	0.05	0.15	60	16	8.5

### Characterization of pectinase

#### Effect of temperature and pH

**Fig. 3:**
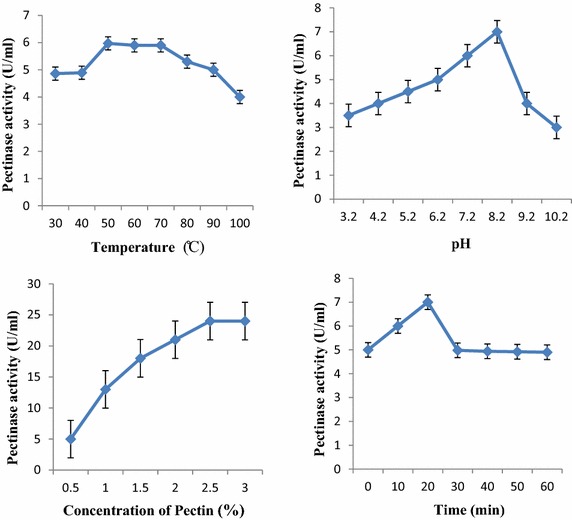
Effect of temperature, pH, pectin concentration, and incubation time on pectinase activity

The enzyme activity was highly influenced by the factors like temperature and pH. In this study, pectinase was found to be highly active at the temperature range of 50–70 °C with the temperature optimum at 50 °C. A slight decrease in the pectinase activity was observed with the increase in temperature. About 82 % of pectinase activity was still retained at 100 °C (Fig. 3). This shows that the pectinase enzyme from *Aspergillus niger* strain MCAS2 was thermostable. In the same way, pectinase enzyme was found active in the pH range 6.2–9.2 with the optimum pH 8.2. This result indicates that the pectinase enzyme is alkaline in nature (Fig. 3).

#### Effect of substrate concentration and incubation time

The pectinase enzyme was found to be optimally active when the assay was carried out in presence of 2.5 % pectin. Further increase in substrate did not show any significant effect on pectinase activity. Similarly, the optimum pectinase activity was observed when the reaction mixture was incubated at 30 °C for 20 min. Although the pectinase activity decreased with increase in incubation time, 74.4 % of pectinase activity was still observed after incubating for 60 min (Fig. 3).

#### Effect of metal ions

The effects of various metal ions (5 mM) on the pectinase activity were investigated at pH 4.2 and 30 °C. The enzyme activity without metal ion was taken as 100 % and relative activity was determined in the presence of metal ions. Metal ions have found to influence the activity of pectinase from *Aspergillus niger* strain MCAS2. Metal ions viz. Cd^+2^, Mg^+2^, Ba^+2^ and Fe^+3^ were found to stimulate the pectinase activity while Na^+^, Pb^+2^, Zn^+2^, and Ca^+2^ inhibited the pectinase activity. The maximum inhibition was observed by calcium ion followed by sodium ion as shown in Table 2.

**Table 2:** Effect of different metal ions on pectinase activity

Metal ions	Activity (%)
None	100
Cd^+2^	147
Mg^+2^	134
Ba^+2^	124
Fe^+3^	113
Pb^+2^	89
Zn^+2^	86
Na^+2^	84
Ca^+2^	59

## Discussion

Pectinase enzyme has been the focus of research for many years due to the potential and wide applications in various industrial processes. An increasing demand of pectinase has rendered the need of exploring microbial strains capable of producing novel pectinases with improved activities. In this study, an attempt has been made to isolate the fungal strains producing pectinase enzyme and to characterize the pectinase enzyme. Out of ten isolated fungal strain, only eight of them were found to be pectinase producer. Among them, strain MCAS2, being the highest producer of pectinase enzyme, was selected for the characterization of pectinase enzyme.

Cultivation of *Aspergillus niger* strain MCAS2 in a liquid medium containing 1 % pectin as a carbon source resulted into maximum production of pectinase at 48 h. The pectinase production time is less than pectinase from other reported fungal strains. Oyeleke et al. (2012) has reported that the production of pectinase after 96 h from *Aspergillus niger*. The pectinase from *Aspergillus flavus* was best produced after 120 h of incubation (Gewali et al. 2007). However, the result was in agreement with the pectinase enzyme produced from *Bacillus subtilis* isolated from soil. It produced the pectinase enzyme after 48 h of incubation (Tripathi et al. 2014). Optimum production of pectinase depends upon the microbial sources as well as the composition of culture media. For the industrial process, shorter fermentation cycle is beneficial from the commercial point of view. Hence, this isolate could have potential biotechnological as well as other industrial application.

Crude pectinase enzyme obtained by the cell culture of *Aspergillus niger* MCAS2 was partially purified by the cold acetone treatment and followed by Sephadex G-75 column chromatography. The activity of partially purified enzyme was increased by 8.5-fold. The molecular mass of isolated pectinase enzyme was 66 kDa as revealed from sodium dodecyl sulphate(SDS)-PAGE. The result is in agreement with the other fungal pectinases such as 63 kDa from *Penicillium frequentans* (Barense et al. 2001), 68 kDa from *Sclerotinia sclerotiorum* (Riou et al. 1992), 60 kDa from *Cochliobolus carbonum* (Scott-Craig et al. 1998), 78 kDa from *Aspergillus tubingensis* (Kester et al. 1996), and 74 kDa from *Fusarium* o*xysporum* sps. (Di Pietro and Roncero 1996). De Vries and Visser (2001) have reported the molecular mass of pectinase in a range of 35–80 kDa. This range is in agreement with the apparent molecular mass of the pectinase enzyme produced by *Aspergillus niger* strain MCAS2.

The partially purified pectinase from *Aspergillus niger* strain MCAS2 is significantly active over the broad range of temperature range 50–70 °C and pH 6.2–9.2 with optimum temperature and pH 50 °C and 8.2 respectively. These results indicate that the pectinase enzyme was thermostable and alkaline in nature. These properties make the enzyme advantageous in various industrial processes that employ high temperature and pH. The fungal pectinase enzymes, especially from *Aspergillus* species, are active in the range of 30–50 °C. Pectinase from *Aspergillus* species have been reported to inactive due to denaturation at temperature above 50 °C (Galiotou-Panayotou et al. 1997). However, the result obtained is in agreement with the pectinase enzyme produced by *Penicillium chrysogenum*, and a thermophilic fungus *Aspergillus fumigatus* which showed that the optimum pectinase activity at 50 °C (Banu et al. 2010; Phutela et al. 2005). Similarly pectinase enzyme from *Aspergillus niger* URM4645 is active at 50–80 °C and *Thermomucorindicae_seudaticae* N31 at 60 °C (Maciel et al. 2011; Martin et al. 2010).

The pectinase enzyme retained 60–90 % activity over the range 6.2–9.2. The similar pectinase enzyme with similar pH profiling have been reported from *Aspergillus niger* URM4645 over the wide pH range (3.5–11.0) (Maciel et al. 2011). Contrary to the result obtained in this study, the pectinase enzyme from *Aspergillus flavus*, *Aspergillus niger*, and *Botrytis cinerea* were active at pH 4.2 (Gewali et al. 1994, 1997, 2007). Fahmy et al. (2008) reported that the pectinase from *Aspergillus niger* has optimum activity at pH 5.0. Similarly, Pectinase from *Penicillium viridicatum* was optimally active at pH ranging from 5.0 to 8.5 (Silva et al. 2002). The fungal pectinase especially from *Aspergillus* species are active in pH range of 4.0–6.0 i.e. the pectinase enzyme is acidic in nature (Tutobello and Mill 1961). The previous reports have shown that alkaline pectinase are mostly produced by bacteria especially *Bacillus* species (Kumar and Sharma 2012; Sonnotel and Nigam 2002; Sonia Ahlawat et al. 2008). The pectinase enzyme from *Bacillus pumilus* dcsr1, *Bacillus stearothermophilus*, *Paenibacillus xylanolyticusm*, *Bacillus halondurans* M29 were shown to be active at high temperature and pH (Sharma and Satyanarayan 2006; Karbassi and Vaughn 1980; Giacobbe et al. 2014; Mei et al. 2013). This thermostability may be attributed to the cystein residue present in the amino acid sequence as observed in pectinase from *Bacillus licheniformis* (Singh et al. 2012). The presence of cystein residue in not only provides the thermostability due to the formation of disulfide bond but also due to strong hydrophobic effect (You et al. 2010). Such residue may be present in the pectinase enzyme isolated from *Aspergillus niger* strain MCAS2 that confers the thermostability to the enzyme. Temperature and pH stability are the most important characteristics of a biocatalyst for their use in industrial applications. So, this fungal pectinase could be the best alternative to bacterial thermostable alkaliphilic pectinase and may find its use in degumming and retting of fiber crops, pulp and paper industries, and pretreatment of pectic waste water from fruit juice industries.

Incubation of pectinase with different concentration of pectin indicated that the enzyme showed maximum activity of 24 U/ml at 2.5 % pectin concentration. Further increase in pectin concentration had no effect in pectinase activity. At high substrate concentration all the enzymes are occupied with substrate so no more substrate can be catalyzed. The maximum enzyme activity was obtained at incubation time of 20 min which can be utilized in the industrial sector for quick product recovery. The metal ions play an important role on activity and stability of enzyme. The pectinase enzyme was strongly inhibited by Ca^+2^ and fairly by Na^+^, Pb^+2^, and Zn^+2^. This correlates with the finding form Yadav et al. that Ca^+2^, Zn^+2^ and Mg^+2^ inhibited the pectinase activity of *Aspergillus flavus* MTCC7589 at 1.0 mmol/l concentration (Yadav et al. 2008). However, the result is in contrary to pectinase enzyme from *Bacillus* sp. KSMp576, *Penicillium chrysogenum*, and mango peel that Ca^+2^ ions found to enhance the pectinase activity (Sonnotel and Nigam 2002; Yadav et al. 2008; Mehrnoush et al. 2012). The isolated pectinase activity was highly activated by Cd^+2^ (more than 50 %) followed by Mg^+2^, Ba^+2^ and Fe^+3^. Whereas, the pectinase activity from *Penicillium chrysogenum* was inhibited by 5 mM Mg^+2^ and Ba^+2^ ions (Banu et al. 2010). This suggests that the requirement of metal ions for the pectinase activity vary depending upon their sources.

## Conclusion

Thermostable and alkaline pectinase from *Aspergillus niger* strain MCAS2 was isolated from Manaslu Conservation Area, Gorkha district, Nepal. This pectinase enzyme with high stability at temperature and pH can be used for various industrial applications including extraction and clarification of fruit juices, processing of cotton fabrics in textile industries, waste water treatment and maceration of tea leaves.

## Abbreviations

ITS: internal transcribed spacer
MCA: Manaslu Conservation Area
SDS-PAGE: sodium dodecyl sulfate–polyacrylamide gel electrophoresis
kDA: kilo dalton
